# Risk Factors for Atelectasis or Pneumomediastinum After Robot-Assisted Partial Nephrectomy

**DOI:** 10.7759/cureus.20383

**Published:** 2021-12-13

**Authors:** Fumiakira Yano, Satoru Kira, Nobuhiro Takahashi, Norifumi Sawada, Hiroshi Nakagomi, Tatsuya Ihara, Masayuki Takeda, Takahiko Mitsui

**Affiliations:** 1 Urology, University of Yamanashi Graduate School of Medical Sciences, Chuo, JPN

**Keywords:** retroperitoneal approach, body mass index, robot-assisted partial nephrectomy, pneumomediastinum, atelectasis

## Abstract

Purpose

Several complications of robot-assisted partial nephrectomy (RAPN) have been reported; however, there are limited data on thoracic findings and complications. We investigated the risk factors for atelectasis or pneumomediastinum after robot-assisted partial nephrectomy.

Methods

This retrospective cohort study included 84 consecutive patients who underwent robot-assisted partial nephrectomy with the da Vinci Si System and the AirSeal^TM^ Insufflation System. Based on chest radiography findings obtained postoperatively in the operating room, patients with and without atelectasis or pneumomediastinum were categorized into Groups A and B, respectively. Patient characteristics (age, sex, body mass index (BMI), RENAL nephrometry score, tumor size, and surgical approach) and perioperative outcomes (total operative time, console time, warm ischemic time, and estimated blood loss) were compared using the Mann-Whitney U test and chi-square test. A multivariate logistic regression analysis was performed to identify the risk factors associated with atelectasis or pneumomediastinum.

Results

Groups A and B included 31 and 53 patients, respectively. Although the rate of the retroperitoneal approach was significantly higher in Group A than in Group B, the other parameters and perioperative outcomes did not differ. The multivariate logistic regression analysis showed that the retroperitoneal approach and high body mass index were risk factors for atelectasis or pneumomediastinum after robot-assisted partial nephrectomy. However, these abnormal findings disappeared spontaneously without requiring postoperative treatment.

Conclusions

The retroperitoneal approach and high body mass index may be risk factors for atelectasis or pneumomediastinum after robot-assisted partial nephrectomy.

## Introduction

As a minimally invasive renal surgery, robot-assisted partial nephrectomy (RAPN) for small renal tumors is being rapidly used worldwide because the three-dimensional high-resolution vision and flexible forceps enable surgeons to perform precise procedures for partial nephrectomy [1,2]. Compared with open or laparoscopic partial nephrectomy, RAPN has superior surgical outcomes [3,4].

As with several laparoscopic renal surgeries, RAPN has been performed under pneumoperitoneum using carbon dioxide. Pneumoperitoneum allows decreased venous blood loss but results in insufflation-related complications such as subcutaneous emphysema, pneumothorax, and pneumomediastinum. In general, routine chest radiography is performed immediately after abdominal surgeries in the operating room to check for abnormal thoracic findings. Although most cases with abnormal findings on routine chest radiography after RAPN or laparoscopic renal surgeries are asymptomatic during the postoperative course, even severe thoracic complications, including pneumothorax or pneumomediastinum related to laparoscopic surgery, may rarely cause life-threatening conditions [5,6]. While several papers have reported that these fatal thoracic complications related to laparoscopic renal surgery are rare, limited data are available on thoracic complications related to RAPN [7,8].

Therefore, we aimed to investigate the incidence of atelectasis or pneumomediastinum on chest radiography after RAPN and identify the risk factors for these abnormal findings.

## Materials and methods

Study design and population

This retrospective cohort study included 84 consecutive patients who underwent RAPN for T1 renal tumors at the University of Yamanashi Hospital between August 2016 and March 2019. The Regional Ethics Committee of the University of Yamanashi for Epidemiological Studies (Institutional Review Board Approval No. 2136) approved the study protocol. We received ethical approval to use an opt-out methodology, and the need to obtain written informed consent from patients was waived. According to the routine chest radiography findings after RAPN in the operating room checked by a reviewer (FY) blinded to all clinical data, patients with and without atelectasis or pneumomediastinum were allocated to Groups A and B, respectively. Data on the following patient characteristics were collected: age, sex, body mass index (BMI), tumor size, surgical approaches, and the RENAL nephrometry score [9]. Surgical outcomes consisted of the total operation time, console time, warm ischemic time, estimated blood loss, and positive surgical margin rate.

Surgery and chest radiography

RAPN was performed by five surgeons (SK, TM, NS, HN, and TI), as previously described [10]. We used the AirSeal^TM^ Insufflation System (CONMED Japan KK, Tokyo, Japan). Briefly, all participants underwent RAPN for renal tumors using the da Vinci Si Surgical System (Intuitive Surgical Inc., Sunnyvale, CA, USA) in all cases. The choice between the transperitoneal or retroperitoneal approaches depended on tumor location, past history of abdominal surgery, or surgeon preference. Four robotic arms and one or two assistant ports were used under the same port place in each approach. After assessing the renal tumor using a robotic ultrasound probe (ProART, BK Medical, Peabody, MA, USA), the tumor was excised with cold scissors under the total clamp of the main renal artery. After tumor excision, an inner running suture with 3-0 V-loc (COVIDEN Japan, Inc., Tokyo, Japan) was performed. Then, the renal artery was unclamped, and renorrhaphy was performed with 2-0 V-loc. Finally, a TachoSil tissue sealing sheet (CSL Behring, Inc., Tokyo, Japan) was placed on the surfaces of the sutured kidney parts. After suturing the skin of the port place, the patients were moved from the lateral decubitus position to the supine position. Then, a routine chest radiograph was obtained under general anesthesia in the operating room.

Statistical analyses

For comparing patient characteristics and surgical outcomes between the groups, the Mann-Whitney U test was used for continuous variables and the chi-square test for categorical variables. To identify the risk factors associated with atelectasis or pneumomediastinum on chest radiography after RAPN, a multivariate logistic regression analysis was performed with the following covariates: age (continuous), BMI (continuous), sex (male/female), surgical approach (transperitoneal/retroperitoneal), and console time (continuous). A p < 0.05 was considered statistically significant. All statistical analyses were performed using SPSS® version 22 (IBM Corp., Armonk, NY).

## Results

Chest radiography findings after RAPN were as follows: atelectasis, 19 patients; pneumomediastinum, nine patients; and atelectasis and pneumomediastinum, three patients. Thus, 31 and 53 patients were assigned to Groups A and B, respectively. All patients in Group A had no symptoms related to these thoracic findings. No patients in both groups underwent open conversion. The rate of the retroperitoneal approach was significantly higher in Group A than in Group B (p < 0.001) (Table 1). Except for surgical approaches, there were no significant differences in patient characteristics and surgical outcomes between the groups (Tables 1, 2). The multivariate logistic regression analysis indicated that the retroperitoneal approach and high BMI were associated with abnormal chest radiography findings (Table 3). During the postoperative course, no patients required surgical intervention for atelectasis or pneumomediastinum, and these thoracic findings disappeared spontaneously.

**Table 1:** Patient characteristics † SD: standard deviation; ‡ BMI: body mass index

	Total	Group A	Group B	p-value
Number of patients	84	31	53	
Age (years), mean (SD^†^)	63.5 (11.9)	67 (11.2)	64 (12.1)	0.50
Sex, n (male/female)	56/28	19/12	37/16	0.42
BMI^‡^(kg/m^2^), mean (SD)	24.1 (3.5)	23.9 (4.2)	23.8 (2.8)	0.23
Approach, n (intraperitoneal/retroperitoneal)	59/25	14/17	45/8	<0.001
Tumor size (mm), mean (SD)	29.5 (11.1)	27.8 (7.7)	30.5 (12.7)	0.66
RENAL score, mean (SD)	7.0 (1.8)	7.3 (1.8)	6.9 (1.8)	0.28

**Table 2:** Surgical outcomes † SD: standard deviation; ‡ EBL: estimated blood loss

	Total	Group A	Group B	p-value
Number of patients	84	31	53	
Total operation time (minutes), mean (SD^†^)	228 (52)	235 (57)	224 (77)	0.39
Console time (minutes), mean (SD)	145 (44.5)	141 ± 44.5	147 ± 44.2	0.42
Ischemic time (minutes), mean (SD)	13 (8.4)	14 ± 10	13 ± 7	0.26
EBL^‡^(mL), mean (SD)	128 (440)	214 (704)	77 (124)	0.73
Surgical margin positive, n	1	1	0	0.19

**Table 3:** Multivariate regression analysis results † CI: confidence interval; ‡ BMI: body mass index

	Odds ratio	95% CI^†^	p-value
Age	1.00	0.96–1.05	0.90
Sex	0.56	0.17–1.89	0.34
BMI^‡^	1.24	1.03–1.49	0.02
Approach	6.68	1.98–22.57	0.002
Console time	0.99	0.98–1.01	0.44
Ischemic time	1.04	0.97–1.11	0.32

## Discussion

The present study showed that 36% (31/84) of the patients undergoing RAPN experienced atelectasis or pneumomediastinum, according to the postoperative chest radiography findings. The multivariate logistic regression analysis indicated that the retroperitoneal approach and high BMI were risk factors for atelectasis or pneumomediastinum after RAPN. However, surgical intervention was not required for these abnormal chest radiography findings in any patient, and these thoracic findings recovered spontaneously.

Previous studies on complications in laparoscopic urologic surgeries demonstrated a low incidence of symptomatic atelectasis necessitating intervention including physiotherapy or intensive care with assisted ventilation [11,12]. A few reports on the various complications of RAPN also demonstrated similar results for atelectasis [13,14]. Although the incidence of atelectasis or pneumomediastinum on chest radiography after RAPN in this study was relatively higher than that reported previously, no intervention or prolonged hospital stay was needed. Because the carbon dioxide in atelectasis or pneumomediastinum after laparoscopic surgeries usually reabsorbs readily, conservative management was usually adopted in these cases. Our results were consistent with those of previous studies regarding symptomatic atelectasis or pneumomediastinum [11-14].

We showed that the retroperitoneal approach was associated with atelectasis or pneumomediastinum on chest radiography after RAPN. Retroperitoneal gas can more easily enter the mediastinum through the diaphragmatic hiatus due to the lack of a subdiaphragmatic peritoneum, resulting in pneumomediastinum [7,8]. In line with this finding, previous studies have reported that the extraperitoneal or retroperitoneal approach in laparoscopic surgeries increased the risk of pneumomediastinum or pneumothorax [7,15]. In this study, the rate of the retroperitoneal approach was higher in patients with pneumomediastinum (Table 4), thus supporting the previously reported mechanism.

The multivariate logistic regression analysis showed that high BMI was also associated with atelectasis or pneumomediastinum on chest radiography after RAPN (Table 5). In general, obesity increases the risk of postoperative atelectasis, especially after surgeries under general anesthesia [16]. We performed RAPN in the lateral decubitus position, regardless of the surgical approach. Because the thoracic cavity opposite to the site of the renal tumor was down during RAPN, atelectasis often occurred in patients with a higher BMI.

The AirSeal^TM^ Insufflation System used in this study is a valveless trocar system that allows smoke evacuation and constant suction while dynamically controlling and stabilizing pneumoperitoneal pressure and maintaining a path for the insertion of endoscopic instruments and sutures [17]. Compared with the conventional insufflation system, the AirSeal^TM^ Insufflation System has been shown to improve perioperative outcomes for RAPN [14,18]. However, no differences in thoracic complications were noted between the AirSeal^TM^ and conventional insufflation systems under the same pneumoperitoneal pressure in these studies [14,18]. On the other hand, the rate of atelectasis or pneumomediastinum was relatively high compared to previous reports [11,12]. We speculated that the reasons were both learning curve and routine chest X-ray at the operating room. Thus, we considered that differences in the insufflation systems were not associated with the incidence of thoracic complications.

This study had some limitations. First, this was a retrospective study with small sample size. Second, detailed findings of chest radiography could not be obtained because of the lack of computed tomography findings after all RAPN procedures. Finally, only a single reviewer checked the chest radiography findings in this study. Thus, studies with a larger number of patients undergoing RAPN in multiple institutions are necessary to confirm our findings.

**Table 4:** Characteristics and surgical outcomes between patients with and without pneumomediastinum † SD: standard deviation; ‡ BMI: body mass index; § EBL: estimated blood loss

	Pneumomediastinum +	Pneumomediastinum -	p-value
Patients, n	12	72	
Age (years), mean (SD^†^)	59.6 (13.3)	64.1 (11.4)	0.22
Sex, n (male/female)	7/5	51/21	0.39
BMI^‡^ (kg/m^2^), mean (SD)	23.3 (3.6)	24.3 (3.4)	0.35
Approach, n (intraperitoneal/retroperitoneal)	1/11	58/14	<0.001
Tumor size (mm), mean (SD)	29.8 (8.0)	29.4 (11.6)	0.86
RENAL score, mean (SD)	7.8 (1.6)	6.9 (1.8)	0.13
Total operation time (minutes), mean (SD)	226 (59)	229 (50)	0.88
Console time (minutes), mean (SD)	136.2 (45.6)	150.5 (44.6)	0.32
Ischemic time (minutes), mean (SD)	19 (11)	16 (8)	0.19
EBL^§^(mL), mean (SD)	60 (92)	140 (469)	0.56

**Table 5:** Characteristics and surgical outcomes between patients with and without atelectasis † SD: standard deviation; ‡ BMI: body mass index; § EBL: estimated blood loss

	Atelectasis +	Atelectasis -	p-value
Patients, n	22	62	
Age (years), mean (SD^†^)	68.6 (8.0)	61.7 (12.4)	0.02
Sex, n (male/female)	13/9	45/17	0.24
BMI^‡^ (kg/m^2^), mean (SD)	26.0 (4.1)	23.5 (2.9)	<0.001
Approach, n (intra/retro)	13/9	46/16	0.18
Tumor size (mm), mean (SD)	27.7 (8.2)	30.1 (12.0)	0.31
RENAL score, mean (SD)	7.1 (1.9)	7.0 (1.8)	0.84
Total operation time (minutes), mean (SD)	242 (50)	223 (52)	0.15
Console time (minutes), mean (SD)	152.0 (44.2)	147.2 (45.2)	0.66
Ischemic time (minutes), mean (SD)	19 (11)	15 (7)	0.09
EBL^§^(mL), mean (SD)	272 (812)	77 (121)	0.07

## Conclusions

Our study showed that 36% of the patients experienced atelectasis or pneumomediastinum on chest radiography after RAPN. Although the rate of the retroperitoneal approach was significantly higher in patients with atelectasis or pneumomediastinum than without it, there were no significant differences in the patient characteristics and surgical outcomes between the groups. The results of multivariate logistic regression analysis indicated that the retroperitoneal approach and high BMI were risk factors for atelectasis or pneumomediastinum after RAPN. However, these cases were usually asymptomatic, and no additional intervention was needed.
